# Supplementary material for: The ARRIVE guidelines 2.0: Updated guidelines for reporting animal research
Source: BMC Vet Res. 2020 Jul 14;16:242. doi: 10.1186/s12917-020-02451-y (PMC7359286; doi:10.1186/s12917-020-02451-y)
Supplement: Supplementary file 1 — Additional file 1. Noteworthy changes in ARRIVE 2.0. This table recapitulates noteworthy changes in the ARRIVE guidelines 2.0, compared to the original ARRIVE guidelines published in 2010. [file 12917_2020_2451_MOESM1_ESM.pdf]

S1 Table: Noteworthy changes in ARRIVE 2.0, compared to the original ARRIVE guidelines published in 2010

ARRIVE 2.0	Original ARRIVE	Reason for change
All items	All items	We reordered items and split them in two sets based on their importance to assess the reliability of the study. There is no ranking within each set, items are ordered logically.
**ARRIVE Essential 10**		
Item 1 – Study design	Item 6 – Study design	We removed the reference to steps taken to minimise the effects of bias (formerly subitem 6b). All information about randomisation is now in item 4 and all information about blinding is now in item 5.
Item 2 – Sample size	Item 10 – Sample size	We clarified that the number of experimental units might be different from the number of animals. Independent replications are now mentioned with the results (item 10) to prevent any confusion with biological replicates.
Item 3 – Inclusion and exclusion criteria	Item 15 – Numbers analysed	We added a new subitem on *a priori* inclusion and exclusion criteria, evidence shows that ad hoc exclusion of data can lead to false positive results [1]. We clarified that the N number in each analysis might be different from the number of animals.<br>We renamed the item to better reflect content.
Item 4 – Randomisation	Item 11 – Allocating animals to experimental groups	All references to randomisation were consolidated in this item for clarity. We reworded the text to include the randomisation procedure which was covered separately in the study design (formerly item 6). We clarified that experimental units are allocated to group, rather than animals.
Item 5 – Blinding	Item 6 – Study design	Blinding was included in the original guidelines as part of the study design (formerly subitem 6b), we have added more text in a new item to highlight its importance and encourage greater specificity.
Item 6 – Outcome measures	Item 12 – Experimental outcomes	We clarified that all outcome measures should be reported, and added a subitem to highlight the need to identify a primary outcome measure for hypothesis-testing studies.<br>We changed the item name to 'outcome measures' because of concerns within the group that the term 'experimental outcomes' could be ambiguous.
Item 7 – Statistical methods	Item 13 – Statistical methods	We removed subitem b about the unit of analysis, which is often poorly understood. Further details are discussed in the supporting E&E document [2].
Item 8 – experimental animals	Item 8 – experimental animals	We clarified the wording and removed examples to streamline the guidelines; further details are discussed in the supporting E&E document [2]. We specified that the details provided needed to be appropriate for the species used.
Item 9 – Experimental procedures	Item 7 – Experimental procedures	We encouraged greater specificity by stating that procedures should be described in enough detail to allow others to replicate them. We removed examples to streamline the guidelines, further details are discussed in the supporting E&E document [2].
Item 10 – Results	Item 16 – outcomes and estimation	We expanded this item to provide more explicit guidance on reporting results. The name of the item was changed from 'outcomes and estimations' to 'results' for clarity and prevent confusion with item 6 – outcome measures.
Item removed	Item 14 – Baseline data	This item overlapped with item 8 – Experimental animals. The two items were combined, with further details provided in the supporting E&E document [2].

ARRIVE Recommended Set		
Item 11 – Abstract	Item 2 – Abstract	We specified that the sex of animals used should be included the abstract, empirical evidence suggests an endemic male bias in biomedical research [3].
Item 12 – Background	Item 3 – Background	We clarified the wording and removed examples to streamline the guidelines, further details are discussed in the supporting E&E document [2].
Item 13 – Objectives	Item 4 – Objectives	We removed a reference to primary and secondary objectives as it would not apply to exploratory studies, and added a requirement to describe the research question, which is relevant to all study types.
Item 14 – Ethical statement	Item 5 – Ethical statement	We removed reference to UK legislation to make this item relevant for an international audience. We added specification of the relevant licence or protocol numbers to provide accountability and promote transparency.
Item 15 – Housing and husbandry	Item 9 – Housing and husbandry	We moved the subitem on welfare-related assessments and interventions to item 16 – Animal care and monitoring. We removed examples to streamline the guidelines, further details are discussed in the supporting E&E document [2].
Item 16 – Animal care and monitoring	Item 17 – Adverse events	We added a new subitem to encourage the reporting of humane endpoints and monitoring, and changed the name of the item to animal care and monitoring to better reflect content.
Item 17 – Interpretation/scientific implications	Item 18 – Interpretation/scientific implications	We removed subitem c "Describe any implications of your experimental methods or findings for the replacement, refinement or reduction (3Rs) of the use of animals in research". This item is not relevant to all animal studies and further details have been provided in the supporting E&E document [2].
Item 18 – Generalisability/ translation	Item 19 – Generalisability/ translation	We simplified and clarified the wording.
Item 19 – Protocol registration	New item	We added a new item on registering key aspects of the protocol. Empirical studies have shown up to 50% of outcomes which are measured are not reported [4]. This selective outcome reporting bias leads to an overstatement of biological effects.
Item 20 – Data access	New item	We added a new item on data access to encourage authors to provide a data sharing statement describing how others can gain access to the data on which the paper is based.
Item 21 – Declaration of interests	Item 20 – Funding	We added a new sub-item on declaring potential conflicts of interest. We added the specification of the role of the funder(s) in the 'design, analysis and reporting of the study'. This information allows the reader to assess any competing interests, and any potential sources of bias.<br>We renamed the item to better reflect content.
Item removed	Item 1 – Title	We removed this item as it provided no specific guidance on what to include in the title.

References

1. Simmons JP, Nelson LD, Simonsohn U. False-positive psychology: undisclosed flexibility in data collection and analysis allows presenting anything as significant. Psychol Sci. 2011;22(11):1359-66. Epub 2011/10/19. doi: 10.1177/0956797611417632. PubMed PMID: 22006061;

2. Percie du Sert N, Ahluwalia A, Alam S, Avey MT, Baker M, Browne WJ, et al. Reporting animal research: Explanation and Elaboration for the ARRIVE guidelines 2.0. PLoS Biol. 2020. doi: 10.1371/journal.pbio.3000411.

3. Beery AK, Zucker I. Sex bias in neuroscience and biomedical research. Neurosci Biobehav Rev. 2011;35(3):565-72. Epub 2010/07/14. doi: 10.1016/j.neubiorev.2010.07.002. PubMed PMID: 20620164; PubMed Central PMCID: PMCPMC3008499

4. Tsilidis KK, Panagiotou OA, Sena ES, Aretouli E, Evangelou E, Howells DW, et al. Evaluation of excess significance bias in animal studies of neurological diseases. PLoS Biol. 2013;11(7):e1001609. Epub 2013/07/23. doi: 10.1371/journal.pbio.1001609. PubMed PMID: 23874156; PubMed Central PMCID: PMC3712913
