# Supplementary material for: The ARRIVE guidelines 2.0: Updated guidelines for reporting animal research
Source: BMC Vet Res. 2020 Jul 14;16:242. doi: 10.1186/s12917-020-02451-y (PMC7359286; doi:10.1186/s12917-020-02451-y)
Supplement: Supplementary file 2 — Additional file 2. Delphi methods and results. Methodology and results of the Delphi study that was used to prioritise the items of the guidelines into the ARRIVE Essential 10 and Recommended Set. [file 12917_2020_2451_MOESM2_ESM.pdf]

S1_Delphi: Delphi methods and results

1 Methods

1.1 Development of the revised checklist

For each subitem of ARRIVE 2010, the NC3Rs summarised the evidence justifying its inclusion in the guidelines and any indication of a need for revision. The ARRIVE Working Group then met for a two-day meeting in November 2017 in London, to review this information, discuss the addition of new items and agree on the strategy to go forward. We agreed to update the guidelines, develop an explanation and elaboration (E&E) document [1] and prioritise the items to facilitate the uptake of the revised guidelines [2]. After the meeting, each item was allocated to at least two members of the group to develop the item's explanation in more detail and refine the item's wording. Further iterations of the checklist were achieved by email discussion within the whole group.

The Delphi exercise was designed to achieve consensus on prioritising items of the ARRIVE guidelines.

The objective was to allocate the 22 items into two or three shortlists with different levels of priority, and relatively even distribution within each set.

1.2 Recruitment of the Delphi expert panel

Ethical approval for this study was obtained from the University of Bristol, Faculty of Science Research Ethics Committee (ID 66625).

The panel consisted of the ARRIVE Working Group and external stakeholders nominated by the Working Group, with suitable expertise on the quality of animal research or its reporting. We aimed to gather a diverse panel of experts, both in terms of field of expertise and geographical location.

Panel members consented to take part by following a link in the invitation email to the first round.

1.3 The Delphi process

There were three iterations of the questionnaire in total [3], and these were managed using the Comet Initiative DelphiManager platform (http://www.comet-initiative.org/DelphiManager/). Data collection took place June to November 2018. Panel members received an email invitation at the start of each round with a link to the online questionnaire. They were allowed three weeks to complete the questionnaire, with email reminders at day 7 and day 14. If they did not respond within the time frame they were excluded from that round, however they were invited to take part in the subsequent rounds of the Delphi.

Each of the 22 items of the revised ARRIVE guidelines was evaluated against the statement:

"How important is this piece of information for assessing the reliability of results in an animal research paper?"

Panel members scored each item on a scale of 1 – 9, where 1 was least important and 9 was most important.

The questionnaire presented in round 1 included free-text fields to provide reasoning for the score given to each item. Individual justifications were collated, summarised and presented to the whole panel in round 2.

In round 2, panel members were asked to provide a justification if their score for a particular item had changed between round 1 and round 2. Similarly, this information was summarised and presented to the whole panel in round 3.

Following rounds 1 and 2, the scores for each item were analysed and a structured summary consisting of a histogram showing the dispersal of the scores in the entire panel was prepared. This summary was presented with a new iteration of the questionnaire at the next round, where panel members were asked to re-score the items. In round 2 and 3, panel members' own scores from the previous round were also displayed for each item.

To encourage a wider dispersal of scores, in the final round (round 3) panel members were asked to follow two rules while scoring items:

- to score no more than ten items in the top range (7 – 9)
- to score no fewer than six items in the bottom range (1 – 3)

In the final dataset we excluded data entries which had not followed these rules, allowing for a deviation of ±1 item in each range.

1.4 Addition of new ARRIVE items

In the first round of the Delphi, we asked the panel to suggest new items that they believed should be included in the revised guidelines. The threshold for inclusion was defined *a priori*; for a new item to be considered, it would have to be suggested by at least 10% of the panel. The panel also had the opportunity to provide general feedback at the end of the survey.

1.5 Criteria for allocating items to sets

The plan to achieve consensus was defined *a priori* and two options were considered. The first option was to allocate the items in three sets, based on each item's median score and a minimum of 70% of the panel scoring the item within the same range. Score ranges were defined as follows:

- top range (7 – 9)
- middle range (4 – 6)
- bottom range (1 – 3)

Should the panel fail to reach agreement using the first option, the second option was to allocate the items in two sets and allocate items with a median score of 7 or above and an agreement level greater than 70% to the first set, and all other items to the second set.

Once data collection was completed, the ARRIVE Working Group met via videoconference to review the results and discuss the allocation of items into sets. As only 10 of 22 items reached the predefined agreement consensus of 70% (see supplementary information S1 Data), the second option was used to allocate items into two sets.

2 Results

2.1 Composition of the Delphi expert panel

One hundred experts were invited to participate in the Delphi exercise, 73 accepted the invitation and 71 participated in the final round (see Figure 1). Ten data entries, which did not follow data dispersal rules were excluded, 61 data entries were therefore included in the final score analysis.

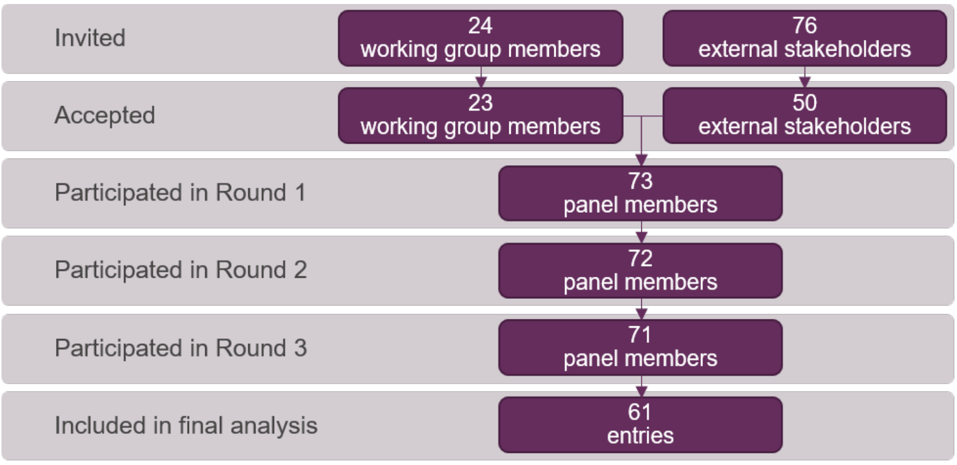

Figure 1. Delphi panel included at each stage of the Delphi exercise.

Demographics of the panel are presented in Table 2.

2.2 Suggestions for new items and feedback on existing items

18 panel members suggested a total of 31 new items (see supplementary information S3 – Delphi data). No new item suggestion met the 10% threshold for inclusion in the revised guidelines.

Feedback on the wording of existing items was considered by the working group in the drafting of the revised items and the drafting of the accompanying E&E document.

Feedback from the Delphi panel indicated that the item on number analysed was misunderstood and confused with the item on sample size. For clarity, the item on number analysed was incorporated to the item on inclusion and exclusion criteria in further iterations of the guidelines. This reduced the number of items to 21.

2.3 Scores for each Delphi round

The scores assigned to each item in rounds 1, 2 and 3 are shown in Figure 2.

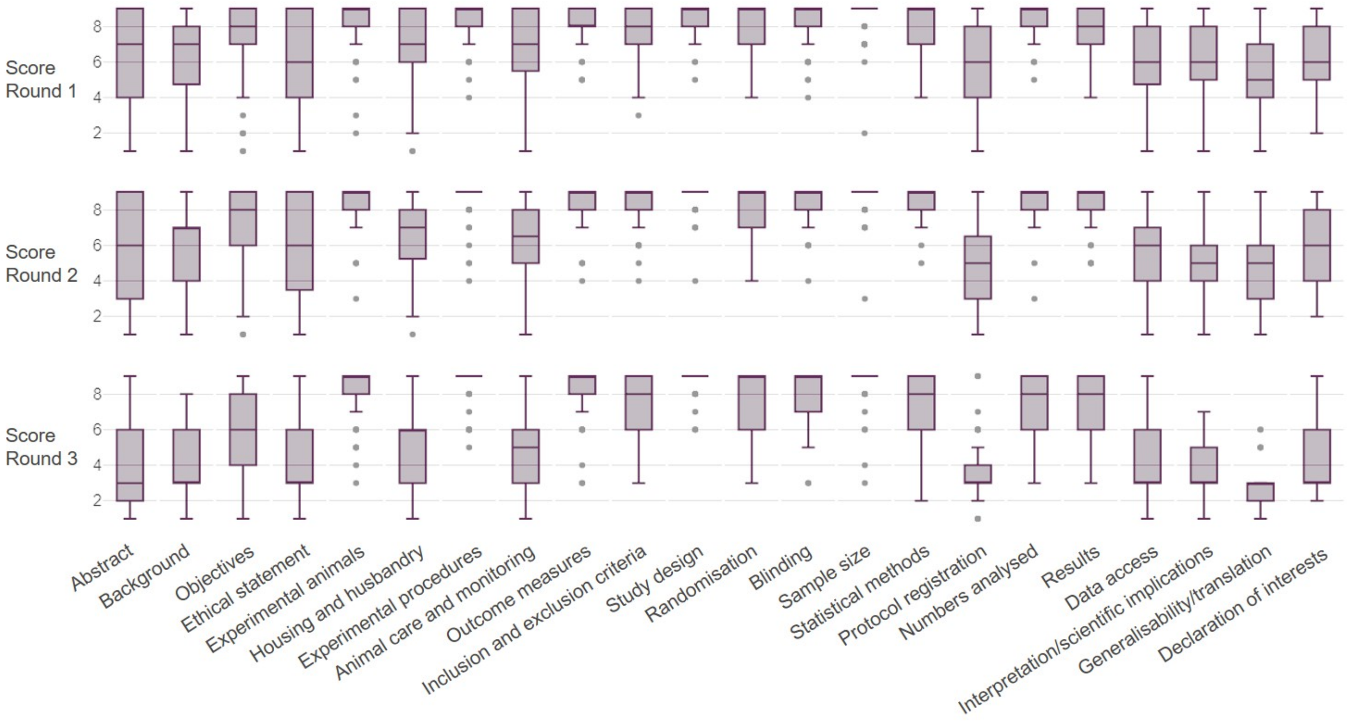

Figure 2. Item scores for each of the three Delphi rounds. Box and whisker plots of the panel members' scores for the 22 items. Round 1: n=71-73, round 2: n=70-71, round 3: n=61, the exact sample size for each item in each round is provided in supplementary information S3 – Delphi data. Data plotted as median, interquartile range, minimum, maximum and outliers using https://www.displayr.com/. Raw data available at https://osf.io/8xjdr/.

2.4. Allocation of items to sets

The allocation of items into sets is presented in Table 1. Eight items were shortlisted based on the *a priori* criterion (score in the top range and over 70% agreement within the panel). Three further items scoring in the top range were added to the shortlist following discussion within the working group.

Note that 11 items were allocated to set 1 but the combination of inclusion and exclusion criteria and numbers analysed in subsequent iterations of the guidelines reduced that number to 10 shortlisted items.

Set	Item	Score	% scores in the top range	Reasoning
Set 1	Study design	9 (9-9)	95	All items met pre-defined threshold (70%) for Set 1.
	Sample size	9 (9-9)	92	
	Experimental procedures	9 (9-9)	87	
	Outcome measures	9 (8-9)	85	
	Experimental animals	9 (8-9)	84	
	Blinding	9 (7-9)	77	
	Randomisation	9 (6-9)	70	
	Statistical methods	8 (6-9)	72	
	Numbers analysed	8 (6-9)	69	Median score in the top range.
	Inclusion and exclusion criteria	8 (6-9)	66	
	Results	8 (6-9)	59	
Set 2	Objectives	6 (4-8)	33	Median score outside the top range.
	Housing and husbandry	6 (3-6)	21	
	Animal care and monitoring	5 (3-6)	10	
	Ethical statement	3 (3-6)	20	
	Abstract	3 (2-6)	16	
	Data Access	3 (3-6)	11	
	Background	3 (3-6)	8	
	Protocol registration	3 (3-4)	7	
	Interpretation/Scientific implications	3 (3-5)	2	
	Declaration of interests	3 (3-6)	2	
	Generalisability/Translation	3 (2-3)	0	

Table 1. Allocation of the 22 items into two sets. Scores are displayed as median and interquartile range (IQR), n=61.

Primary country of work	
UK	27
USA	11
Canada	7
Brazil	5
Australia	3
China	4
Germany	2
Switzerland	2
The Netherlands	2
Argentina	1
Belgium	1
India	1
Japan	1
Korea	1
Nigeria	1
Norway	1
South Africa	1
South Korea	1
Sri Lanka	1

Sector of work	
Academia	45
Not-for-profit	7
Industry	6
Publishing	6
Government	5
Funding body	1
Media	1
Educator	1
Contract research company	1

Years of relevant experience	
1 - 10	13
11 - 20	27
21 - 30	22
31 - 40	8
41 - 50	3

Career level	
Principle Investigator	32
Senior Staff	5
Associate Professor	4
Director	4
Staff Scientist	3
Editor	2
Executive Editor	2
Postdoctoral scientist	2
Senior Editor	2
Associate Director	1
Associate Editorial Director	1
Editorial Director	1
Head of Department	1
Head of Laboratory and Research	1
Head of Policy	1
Lab Animal Facility Director	1
Manager of Clinical Phenotyping Core	1
Managing Editor	1
Masters/PhD student	1
Mid-management	1
Policy analyst	1
Science Administrator	1
Senior Manager	1
No answer	3

Professional role	
In vivo researcher	32
Journal editor	20
Statistician	6
Professor	5
Systematic review/ meta-researcher	4
Veterinarian/ assistant veterinarian	4
Director of a lab animal facility	3
Former *in vivo* researcher	3
Educator	2
Project manager	2
Publisher	2
Reviewer of *in vivo* research	2
Associate editorial director	1
Clinician	1
Director of research policy for a research funder	1
Director of research quality for a research funder	1
Director of standards	1
Evidence synthesis specialist	1
Head of experimental design for a research funder	1
Mathematical biologist	1
Op-ed editor	1
Policy analyst for a research funder	1
Preclinical bioresearch quality & compliance	1
Researcher using *in vitro* methods and human subjects	1
Science administrator	1
Scientific director	1
Scientist/ manager	1
Secretary of a 3Rs centre	1
Senior program officer (science policy)	1

Table 2. Demographics of Delphi respondents (n = 73). Note that the total number of professional roles exceeds the number of panel members as they could select more than one role.

References

1. Percie du Sert N, Ahluwalia A, Alam S, Avey MT, Baker M, Browne WJ, et al. Reporting animal research: Explanation and Elaboration for the ARRIVE guidelines 2.0. PLoS Biol. 2020. doi: 10.1371/journal.pbio.3000411.

2. Percie du Sert N, Hurst V, Ahluwalia A, Alam S, Altman DG, Avey MT, et al. Revision of the ARRIVE guidelines: rationale and scope. BMJ Open Science. 2018;2(1). doi: 10.1136/bmjos-2018-000002.

3. Boulkedid R, Abdoul H, Loustau M, Sibony O, Alberti C. Using and reporting the Delphi method for selecting healthcare quality indicators: a systematic review. PLoS One. 2011;6(6):e20476. Epub 2011/06/23. doi: 10.1371/journal.pone.0020476. PubMed PMID: 21694759; PubMed Central PMCID: PMCPMC3111406
