# Supplementary material for: The ARRIVE guidelines 2.0: Updated guidelines for reporting animal research
Source: BMC Vet Res. 2020 Jul 14;16:242. doi: 10.1186/s12917-020-02451-y (PMC7359286; doi:10.1186/s12917-020-02451-y)
Supplement: Supplementary file 4 — Additional file 4. Road testing methods and results. Methodology used to road test the revised ARRIVE guidelines and E&E (as published in preprint) and how this information was used in the development of ARRIVE 2.0. [file 12917_2020_2451_MOESM4_ESM.pdf]

S1_Road_testing: ARRIVE guidelines road testing methods and results

1 Purpose

The road testing was designed to test the revised ARRIVE checklist and accompanying Explanation and Elaboration document (E&E) published as preprints on BioRxiv. The purpose was to identify whether authors of animal research manuscripts understood the wording of each item of the revised guidelines, and whether the E&E helped them identify the information to be reported for each ARRIVE item.

2 Methods

2.1 Participant recruitment

Ethical approval for this study was obtained from the University of Bristol, Faculty of Science Research Ethics Committee (ID 93122). The study was conducted September-November 2019.

Road testing participants were researchers preparing manuscripts which included animal experiments. They were recruited using social media and contacts of the working group and NC3Rs staff. We aimed to recruit a diverse group of 10-15 animal researchers, which is suitable to identify 80-90% of the issues in usability testing [1].

Participants could enter the study if they were preparing a manuscript that included *in vivo* experiments. Participants who completed the process received a £50 / US$60 Amazon voucher.

2.2 The road testing process

Participants were asked to use the preprint version of the ARRIVE guidelines 2019 [2] and the E&E [3] while preparing their manuscript, and to report their experiments in adherence with each of the 21 items of the guidelines. They were asked to complete an ARRIVE 2019 checklist and indicate in which section or on what line number the information related to each item was reported, or provide a reason for not reporting that information.

Upon reception, manuscripts and checklists were anonymised and then reviewed by two members of the ARRIVE Working Group to assess whether reporting was in adherence with the ARRIVE guidelines 2019.

Following manuscript review, each participant was interviewed:

1. to gather feedback on the clarity and usefulness of the guidelines and E&E,
2. to discuss any discrepancy between the information provided in their checklist and the assessment of the information contained in the manuscript,
3. to gather further insight on the reasons for not including specific pieces of information recommended by the guidelines.

3 Results

3.1 Participants

Forty-four researchers showed interest in participating in road testing. Twenty-two researchers confirmed that they met the eligibility criteria, 11 of which completed the road testing process. The 11 participants were based in four different countries and worked in different sectors in a variety of research fields, including cancer biology, pharmacology, neuroscience, immunology, nanomedicine, developmental biology, cardiovascular, welfare and behaviour research. Participants were also at various career stages (see table 1).

Career level/professional role	
Postdoc	4
PhD student	1
Research fellow	1
Vet	1
Senior scientist	1
Principal Investigator	1
Professor	1
Director of Research	1

Primary country of work	
UK	7
USA	2
Belgium	1
Brazil	1

Native English speaker	
Yes	6
No	5

Sector of work	
Academia	7
Industry	1
Government laboratory	2
Non-profit	1

Model organism used	
Mouse	4
Rat	3
Fish	3
Frog	1

Table 1. Demographics of the participants who completed the ARRIVE guidelines road testing (n = 11).

3.2 Overview of the users' feedback and adjustments to the guidelines and E&E

Participants used the guidelines and the E&E in different ways. Some did not read the E&E and used only the checklist, others read the E&E first and then used the checklist and a further group used the checklist and referred to the E&E for help with specific items. Using the checklist prompted nine participants to add information into their manuscript, for seven of the manuscripts, this included information covered in the Essential 10. Anonymised general feedback from each participant is presented in S2 Data.

For a number of ARRIVE items, the information captured in the checklist was clearer than the information provided in the manuscript, or the information was only provided in the checklist but not in the manuscript. Information which authors declined to provide in the manuscript and only disclosed in the checklist generally included things that had not been used or observed during the study, for example that no inclusion and exclusion criteria had been set, no data had been excluded, randomisation or blinding had not been used, assumptions of the statistical approach had not been assessed, adverse events had not been observed or the protocol had not been registered. The checklists also contained information that authors did not include in the manuscript because of space restrictions, because the *in vivo* experiment was a minor component of the study, because they considered the information to be obvious, or because they were unsure of how to incorporate it in the manuscript.

This highlights the importance of publishing a completed ARRIVE checklist with the manuscript as it enables authors to disclose further information that they are not willing to describe, or did not realise was not clear, in the manuscript itself.

Based on the information missing from each manuscript and the reasons discussed with authors, we adjusted the wording of the following items:

- **3. Inclusion and exclusion criteria** – to separate the requirement to report criteria from the requirement to state when they were established, to add that if no criteria were set, this should be stated explicitly (3a), and to specify that if there were no exclusions, this should be stated (3b).
- **4. Randomisation** – to clarify that the method of randomisation relates to how the random sequence was generated (4a) and to specify that uncontrolled confounders should be reported (4b).
- **7. Statistical methods** – to remove a reference to the experimental unit used in statistical test, which confused most users.
- **16. Animal care and monitoring** – to include the signs monitored and clarify what to report if humane endpoints were not set for the study.

We also refined or complemented the explanations in the E&E document to address some of the issues detected and clarify the meaning for each item. Detail of the information missing with justifications for each manuscript and the subsequent adjustments we made to the guidelines and the E&E are presented in S2 data.

References

1. Faulkner L. Beyond the five-user assumption: benefits of increased sample sizes in usability testing. Behav Res Methods Instrum Comput. 2003;35(3):379-83. Epub 2003/11/01. PubMed PMID: 14587545;

2. Percie du Sert N, Hurst V, Ahluwalia A, Alam S, Avey MT, Baker M, et al. The ARRIVE guidelines 2019: updated guidelines for reporting animal research. bioRxiv. 2019:703181. doi: 10.1101/703181.

3. Percie du Sert N, Ahluwalia A, Alam S, Avey MT, Baker M, Browne W, et al. Reporting animal research: Explanation and Elaboration for the ARRIVE guidelines 2019. bioRxiv. 2019:703355. doi: 10.1101/703355.
